# It’s time for a minimum synoptic operation template in patients undergoing laparoscopic cholecystectomy: a systematic review

**DOI:** 10.1186/s13017-022-00411-5

**Published:** 2022-03-17

**Authors:** Niall O’Connor, Michael Sugrue, Conor Melly, Gearoid McGeehan, Magda Bucholc, Aileen Crawford, Paul O’Connor, Fikri Abu-Zidan, Imtiaz Wani, Zsolt J. Balogh, Vishal G. Shelat, Giovanni D. Tebala, Belinda De Simone, Hani O. Eid, Mircea Chirica, Gustavo P. Fraga, Salomone Di Saverio, Edoardo Picetti, Luigi Bonavina, Marco Ceresoli, Andreas Fette, Boris Sakakushe, Emmanouil Pikoulis, Raul Coimbra, Richard ten Broek, Andreas Hecker, Ari Leppäniemi, Andrey Litvin, Philip Stahel, Edward Tan, Kaoru Koike, Fausto Catena, Michele Pisano, Federico Coccolini, Alison Johnston

**Affiliations:** 1 Department of Surgery, Letterkenny University Hospital and Donegal Clinical Research Academy, Donegal, Ireland; 2 EU INTERREG Centre for Personalized Medicine, Intelligent Systems Research Centre, School of Computing, Engineering and Intelligent Systems, Ulster University, Magee Campus, Derry-Londonderry, Northern Ireland; 3 Department of Anaesthesia, Letterkenny University Hospital, Donegal, Ireland; 4 Department of Surgery, College of Medicine and Health Sciences, UAE University, Al-Ain, United Arab Emirates; 5 Government Gousia Hospital, Srinagar, India; 6 John Hunter Hospital and University of Newcastle, Newcastle, NSW Australia; 7 Tan Tock Seng Hospital, Singapore, Singapore; 8 Oxford University Hospitals NHS Foundation Trust, John Radcliffe Hospital. Headley Way, Headington, Oxford, OX3 9DU UK; 9 Poissy/Saint Germain en Laye Hospitals, Poissy-Ile de France, France; 10 Abu Dhabi Police Aviation, HEMS, Abu Dhabi, UAE; 11 Centre Hospitalier Universitaire Grenoble Alpes, Grenoble, France; 12 Division of Trauma Surgery, School of Medical Sciences, University of Campinas (Unicamp), Campinas, Brazil; 13 Hospital of San Benedetto del Tronto (AP), San Benedetto del Tronto, Italy; 14 Department of Anesthesia and Intensive Care, Parma University Hospital, Parma, Italy; 15 Division of General and Foregut Surgery, IRCCS Policlinico San Donato, Department of Biomedical Sciences for Health, University of Milano, Milan, Italy; 16 General and Emergency Surgery, School of Medicine and Surgery, University of MIlano-Bicocca, Monza, Italy; 17 PS_SS Weissach Im Tal, Weissach im Tal, Germany; 18 RIMU/Research Institute at Medical University of Plovdiv, Plovdiv, Bulgaria; 19 Department of Surgery, Attikon General Hospital, National and Kapodistrian University of Athens, Athens, Greece; 20 Riverside University Health System Medical CA and Loma Linda University School of Medicine CA, Riverside, USA; 21 Department of Surgery. Radboud University Medical Centre, Nijmegen, The Netherlands; 22 Department of General and Thoracic Surgery, University Hospital of Giessen, Giessen, Germany; 23 Helsinki University Hospital and University of Helsinki, Helsinki, Finland; 24 Department of Surgical Disciplines, Immanuel Kant Baltic Federal University, Regional Clinical Hospital, Kaliningrad, Russia; 25 Department of Specialty Medicine, College of Osteopathic Medicine, Rocky Vista University, Parker, CO 80134 USA; 26 Department of Surgery, Radboud University Medical Center, Nijmegen, The Netherlands; 27 Kyoto Medical Center, Kyoto, Japan; 28 Bufalini Hospital, Cesena, Italy; 29 Papa Giovanni XXIII Hospital, Bergamo, Italy; 30 General, Emergency and Trauma Surgery Department, Pisa University Hospital, Pisa, Italy

**Keywords:** Laparoscopic cholecystectomy, Synoptic reporting, Operation notes, Patient safety

## Abstract

**Background:**

Despite the call to enhance accuracy and value of operation records few international recommended minimal standards for operative notes documentation have been described. This study undertook a systematic review of existing operative reporting systems for laparoscopic cholecystectomy (LC) to fashion a comprehensive, synoptic operative reporting template for the future.

**Methods:**

A search for all relevant articles was conducted using PubMed version of Medline, Scopus and Web of Science databases in June 2021, for publications from January 1st 2011 to October 25th 2021, using the keywords: laparoscopic cholecystectomy AND operation notes OR operative notes OR proforma OR documentation OR report OR narrative OR audio-visual OR synoptic OR digital. Two reviewers (NOC, GMC) independently assessed each published study using a MINORS score of ≥ 16 for comparative and ≥ 10 for non-comparative for inclusion. This systematic review followed PRISMA guidelines and was registered with PROSPERO. Synoptic operative templates from published data were assimilated into one “ideal” laparoscopic operative report template following international input from the World Society of Emergency Surgery board.

**Results:**

A total of 3567 articles were reviewed. Following MINORS grading 25 studies were selected spanning 14 countries and 4 continents. Twenty-two studies were prospective. A holistic overview of the operative procedure documentation was reported in 6/25 studies and a further 19 papers dealt with selective surgical aspects of LC. A unique synoptic LC operative reporting template was developed and translated into Chinese/Mandarin, French and Arabic.

**Conclusion:**

This systematic review identified a paucity of publications dealing with operative reporting of LC. The proposed new template may be integrated digitally with hospitals’ medical systems and include additional narrative text and audio-visual data. The template may help define new OR (operating room) recording standards and impact on care for patients undergoing LC.

**Supplementary Information:**

The online version contains supplementary material available at 10.1186/s13017-022-00411-5.

## Introduction

Clear decision making and precise operative strategy and documentation are fundamental to optimizing surgical outcomes. Despite implementation of quality improvement programs including digital transformation of many medical systems the surgical operation report remains inadequate, with many inaccuracies and under-reporting of the actual procedure undertaken [1].

Changes to operative documentation were recommended over one hundred years ago by Eugène-Louis Doyen, who advocated the use of cinematography to document and improve outcomes [2]. Fifty years ago, dictation and typing of operative notes was suggested by Stanley-Brown, to address legibility [3] and, more recently, Ballester and colleagues recommended electronic synoptic documentation [4, 5]. Despite the advocacy for better records, change has been slow, in part due to lack of training of surgical trainees in proper documentation of operative procedures [4, 6].

To enhance the accuracy and value of operative documentation international recommended minimal standards have been proposed (Table 1) [7, 8].

**Table 1:** Royal College of Surgeons of Ireland criteria for operative synoptic reporting

1	Date and time
2	Elective/emergency procedure
3	The names of the operating surgeon(s) and assistant(s)
4	The operative procedure carried out
5	The incision
6	The operative diagnosis
7	The operative findings
8	Any problems/complications
9	Any extra procedure performed and the reason why it was performed
10	Details of tissue removed, added or altered
11	Identification of any prosthesis used, including the serial numbers of prostheses and other implanted materials
12	Details of closure technique
13	Post-operative care instructions
14	Signature

Operation notes are fundamental in communicating patient care, resident education, information for surgeons and act as a resource tool for outcome improvement and research [9]. Furthermore, they provide a mechanism for healthcare reimbursement and an improvement in quality of care [10].

There is increasing realization of the value of synoptic operative notes which are templated and procedure specific [11]. Inclusion of archived (and retrievable) video recordings and intra-operative photographs may enhance reporting, not just of surgical procedures but in many areas of medicine [12–14]. As part of change management in surgery physician and patient concerns regarding privacy, data protection, and potential medico-legal exposure need to be addressed [15].

Laparoscopic cholecystectomy (LC) is one of the commonest operations performed globally, with over 1.1 million procedures per annum in the United States alone [16]. To understand the patient’s path to recovery and potential adverse outcomes, which may occur in up to 20% of patients, requires a transparent description of operative findings and procedures is advisable [17].

It has been recognized that operative notes may not, in their current format, adequately represent the actual performed procedure with suboptimal use of intra-operative imaging [12, 18–20]. Many guidelines relating to the management of cholecystitis have been produced, but, to our knowledge, none have dealt with the operative report [21, 22].

Comprehensive reporting systems need to provide insight into a surgeon’s decision-making and facilitate a better understanding of intra-operative difficulties. Few studies have created a comprehensive gallbladder operative reporting template [23]. The aim of this study was to undertake a systematic review of existing operative reporting systems for LC and propose a comprehensive, synoptic operative reporting template for the future.

## Methods

### Search strategy

A search for all relevant articles was conducted using the PubMed version of Medline, Scopus and Web of Science electronic databases in June 2021. The search was conducted using the keywords: laparoscopic cholecystectomy AND operation notes OR operative notes OR proforma OR documentation OR report OR narrative OR audio-visual OR synoptic OR digital. MeSH terms were used to search PubMed and Scopus. Articles from January 1st, 2011, to October 25th, 2021 were chosen to capture current literature.

### Inclusion and exclusion criteria

In order to avoid selection bias, the methods of the systematic review and the inclusion criteria of the study were specified in advance and documented in a protocol which was registered with the PROSPERO database (International Prospective Register of Systematic Reviews) registration number: CRD42021292839. This systematic review followed the Preferred Reporting Items for Systematic Reviews and Meta-Analyses (PRISMA) guidelines [24]. Full text, English language articles reporting studies on operative documentation of patients undergoing LC were included. Systematic reviews, meta-analyses, case reports, editorial comments and letters were excluded, as were studies of paediatric (< 16 years of age) patients or pregnant patients. Citations were exported into Microsoft Excel and duplicates were subsequently removed. The reference sections of reviewed studies were examined for eventual further retrieval of papers not identified by the initial search strategy.

### Study selection and data extraction

Once identified by the search strategy, studies were screened for inclusion initially by title, then abstract and subsequently by full text review. Eligibility assessment was performed independently by two reviewers (NO’C, CM). Disagreements were resolved by consensus and if no agreement could be reached a third reviewer (AJ) was involved.

Two reviewers (NOC, GMC) independently assessed each published study for the quality of study design and risk of bias by using standardized pre-piloted forms incorporating the methodological index for non-randomized studies (MINORS) score [25]. A MINORS score of ≥ 16 out of 24 for comparative and ≥ 10 for non-comparative was considered the standard for inclusion.

A critical evaluation of each study was conducted by the reviewers. The method of the operative note documentation was recorded as handwritten, dictated and typed, or electronically stored. In addition, the use of a template or synoptic report was documented, including any facility for digital archiving and storage of operative photographs or videos. Synoptic operative templates from published data were assimilated into one “ideal” laparoscopic operative report template.

## Results

**Fig. 1:**
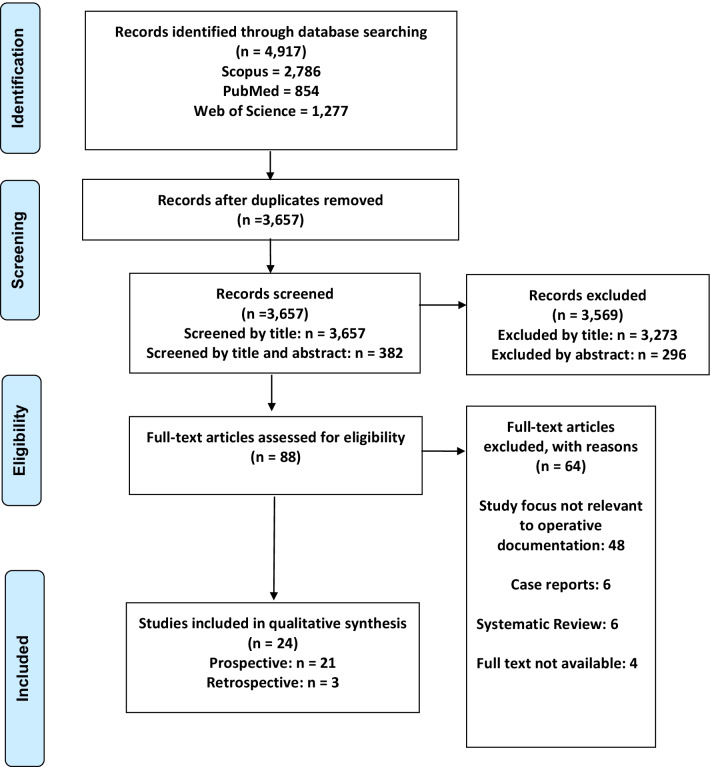
PRISMA flow diagram: identification, review and selection of articles included in the systematic review

A total of 3567 articles were reviewed. Following qualitative assessment by MINORS grading 24 studies were selected spanning 14 countries and 4 continents. Twenty-two studies were prospective. Reference section review yielded one additional paper by Harvey et al. which was included in the final data synthesis making a total of 25 studies (Fig. 1) [9, 12, 13, 18–20, 23, 26–43].

A holistic overview of the operative procedure documentation was reported in 6/25 studies (Table 2).

**Table 2:** Studies undertaking comprehensive operative note review

Author	Year	Country	Study design	Main selective area
Deal	2018	USA	Prospective	Synoptic operative reporting
Harvey	2007	UK	Prospective	Synoptic operative reporting
Shaikh	2019	Pakistan	Prospective	Synoptic operative reporting
Thomson	2016	UK	Prospective	Synoptic operative reporting
Wauben	2011	The Netherlands	Prospective	Operative note accuracy
Wauben	2013	The Netherlands	Prospective	Operative note accuracy

A further 19 papers dealt with selective surgical aspects such as the inclusion of audio-visual tools and scoring of biliary anatomy (Table 3).

**Table 3:** Studies with selective surgical documentation review

Author	Year	Country	Study design	Main selective area
Balla	2018	Italy	Prospective	Grade of biliary anatomy injury
Bolivar-Rodriguez	2018	Mexico	Prospective	Imaging biliary anatomy
Booij	2018	The Netherlands	Retrospective	Operation note accuracy
Buddingh	2011	The Netherlands	Prospective	Biliary anatomy documentation
Cho	2017	Korea	Prospective	Grading of biliary anatomy injury
Eryigit	2020	The Netherlands	Prospective	Audio and video recording of cholecystectomy
Fingerhut	2013	The Netherlands	Prospective	Common bile duct injury grading system
Griffiths	2019	UK	Prospective	Grade of cholecystitis
Lam	2014	Australia	Prospective	Imaging biliary anatomy
Loukas	2018	Greece	Prospective	Procedure video documentation
Nassar	2020	UK	Prospective	Pre-operative risk scoring
Sakowska	2016	New Zealand	Prospective	Digitalized hospital workflow system
Sanford	2014	USA	Prospective	Imaging biliary anatomy
Sebastian	2021	Poland	Prospective	Imaging biliary anatomy
Siada	2019	USA	Retrospective	Grade of cholecystitis
Sugrue	2015	Global	Prospective	Grade of cholecystitis
Sugrue	2019	Global	Prospective	Grade of cholecystitis
Tullavardhana	2016	Thailand	Retrospective	Video and photo documentation of anatomy
Vivek	2014	India	Prospective	Grade of operative difficulty

An operative template reporting tool was reported in 6 studies [9, 13, 18, 20, 31, 42]. Deal and colleagues reported the use of a dictated operative template which incorporated 42 data fields including patient and operating team identifiers, pre-operative assessment, intra-operative findings, intra-operative complications, procedures, the use of intra-operative cholangiography (IOC), use of drains and wound closure technique [9]. They did not include post-operative instructions or mention the surgeon’s signature. The study by Harvey et al. reported 14 items which were subdivided and contained expanded prompts within the template [31]. These included documentation of operative urgency, patients’ admission status and indication for surgery. Booij et al. developed a template of 33 operative details to analyze patients’ referrals to a single centre with common bile duct (CBD) injuries post LC [20].

Eryigit et al. used an 11-point operative template including 8 sub-headings related to visualisation of trocar introduction and removal [13].

In a comparison of 125 video recordings and operative notes, Wauben et al. reported 6 key steps of the LC based on the 2006 guideline from the Dutch Surgical Society (DSS) [18, 44]. These included: (1) trocars insertion under direct vision; (2) gallbladder’s condition; (3) safety critical view (CVS); (4) clips placement; (5) liver haemostasis, and (6) trocar removal under vision. CVS was defined as completely unfolding Calot’s triangle with mobilizing of gallbladder neck from its bed on the liver before clipping and transecting the cystic artery and duct. In a more recent publication, Wauben et al. compared different surgeons’ LC reports against a list of 45 items in the operative template, including 15/45 items detailing trocar size, location and removal [42].

Thomson et al. performed an audit of 130 LC operative notes to determine compliance with Royal College of Surgeons (RCS) and DSS reporting standards. The authors then created a synoptic template containing 56 items for documentation (with narrative options) before prospectively evaluating a further 128 templated LC reports for completion of the audit cycle [23].

Very few studies used hand written reports, up to 70% of which were illegible in a study by Baigrie from 1994 [3]. Digital archiving LC systems have not been published to our knowledge but are appearing on the web https://www.touchsurgery.com/ [45]. Links to billing were mentioned in one study and there is an opportunity to enhance coding and billing through an accurate procedure recording [10].

### Completion rates

In a multi-institutional evaluation of synoptic operative reports (SORs) versus dictated operative reports (DORs) in 35 patients undergoing LC, Deal and colleagues reported completion rates of 99.7% for SORs versus 76% for associated DORs [9]. Moreover, 87% of surveyed surgeons in the study indicated a preference for the synoptic format. A brief narrative comment was added in 48.5% of cases.

Thomson et al. showed a significant improvement in documentation rates for procedural data upon introduction of an SOR for LC in a three-hospital NHS Trust, including operative time (82% SOR vs. 25% DOR), operative setting (95% SOR vs. 3% DOR), complications (83% SOR vs. 49% DOR), name of surgeon (99% SOR vs. 93% DOR) and signature (96% SOR vs. 88% DOR), but a decrease in documentation of the procedure date (89% vs. 99%) [23]. The authors also found a significant positive correlation between the surgical experience level and DOR completion rates (*p* < 0.0001), although the correlation was no longer significant following SOR introduction.

In a prospective series of 25 consecutive LC performed in a single institution, Shaikh et al. demonstrated a 79% completion rate in SORs versus 25% in DORs [36].

### Intra-operative image recording

Intra-operative photography during LC has been used to document the CVS. Adequacy of such photography in achieving the CVS was reviewed by two expert observers in a prospective audit of 100 consecutive LCs [19]. The measured rate of an adequate CVS was 52% and 45%, respectively. This raises the question of need for artificial intelligence or machine learning algorithms to help in assess completeness. Sanford and colleagues proposed a method of “doublet” photography which combines both anterior and posterior imaging of the CVS [35]. In this study of a series of 28 elective LCs, photographs of anterior, posterior and doublet view were rated by two independent surgeons. Anterior or posterior images alone received significantly lower ‘satisfactory’ ratings than doublet views (76.8% vs. 96.4%, *p* = 0.02). Buddingh et al. found IOC to be more conclusive than photography of the CVS for documentation of biliary anatomy, with 57% of IOCs conducted in 63 procedures deemed conclusive by blinded experts versus 25% for photographs of the CVS for the same procedures [28]. Eryigit et al. reported that video documentation of LCs adequately depicted surgical steps in 1005/1089 (92.3%) video observations compared to 849/1089 (78%) in operative notes (*p* < 0.001) [13]. The addition of audio recordings resolved some discrepancies between video recordings and operative notes, resulting in a drop in discrepancy from 23% with audio adjustment to 11.8% without (*p* < 0.001).

The integration of SORs into a hospital medical record system was addressed by Sakowska et al. [34]. These authors reported uptake of SORs for LCs rose from under 20% in the first month to 100% within the second month after introduction and remained > 90% for the next 7seven months. SORs were immediately available when patients arrived in the recovery room and reached the electronic health record of the hospital within a median time of 5 min (IQR 3–8 min, *n* = 425), compared to a median time of 2two days for traditional DORs (IQR 1–5 days, *n* = 174).

### Scoring systems

Documentation of scoring/grading systems were reported in six papers, three relating to gallbladder scoring, two to bile duct injury and one to the development of a pre-operative risk score [26, 30, 33, 38, 39, 43]. Operative difficulty was the subject of four publications which focused on predictive scoring systems for difficulties encountered during LC. Griffith et al. aimed to validate a difficulty grading system (Nassar scale) by testing its applicability in two large databases [30]. As the difficulty grade increased from 1 to 4, they found an increase in the median length of stay from 0 to 4 days, as well as an increase in 30-day complication rates from 7.6 to 24.4% (both *p* < 0.001).

In a prospective, multi-institutional, web-based study of over 500 LC, Sugrue et al. reported a laparoscopic to open conversion rate of 14.1% and showed a positive correlation between increased scores on the G10 gallbladder scoring system and conversion to open surgery, with 33% of operations with G10 scores of ≥ 5 being converted to open (*p* < 0.001) [39]. A pre-operative risk score based on 8 independent predictors of difficulty was used to classify low, medium and high risk patients in a study by Nasser et al. [33]. In this study the proportion of difficult operations was 11.0% in low-risk, 31.1% in medium risk and 80.0% in high risk patients. On external validation, the score returned an area under the ROC curve of 0.789 (95% CI 0.773–0.806, *p* < 0.001).

Two of the included papers dealt with biliary duct injury (BDI) scoring systems, specifically the ATOM scoring system proposed by Fingerhut et al. [43]. Balla et al. reviewed 26 patients who presented with BDI to a single institution and concluded that ATOM classification included every aspect of each case of BDI within their study, whereas 5 other main classifications lacked at least 1 relevant injury detail [26].

**Fig. 2:**
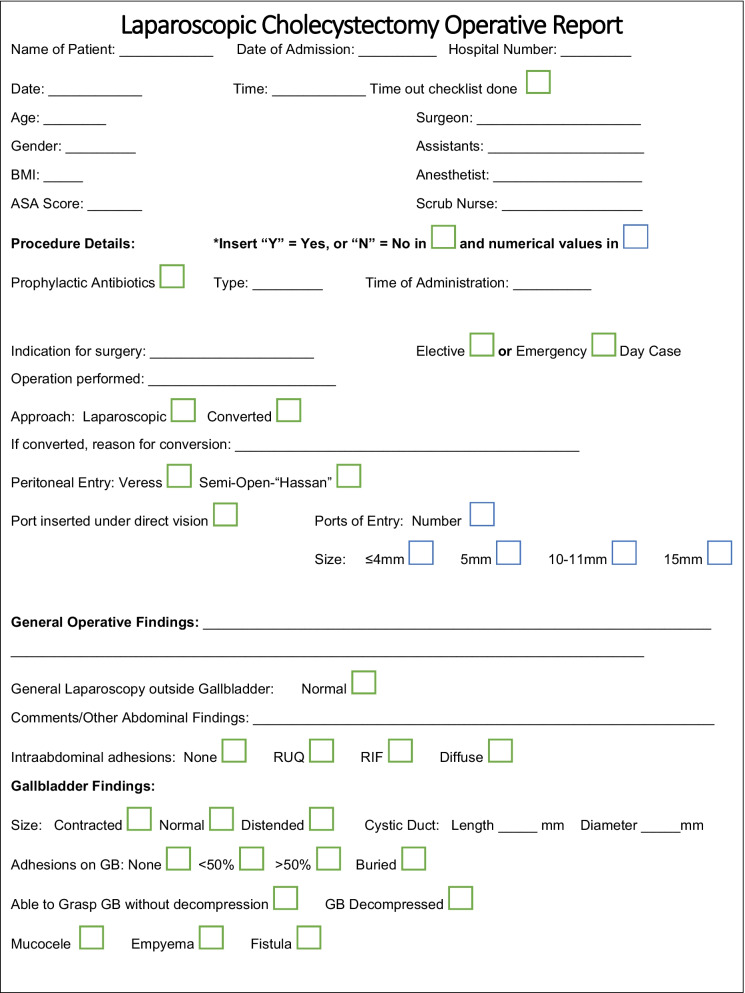

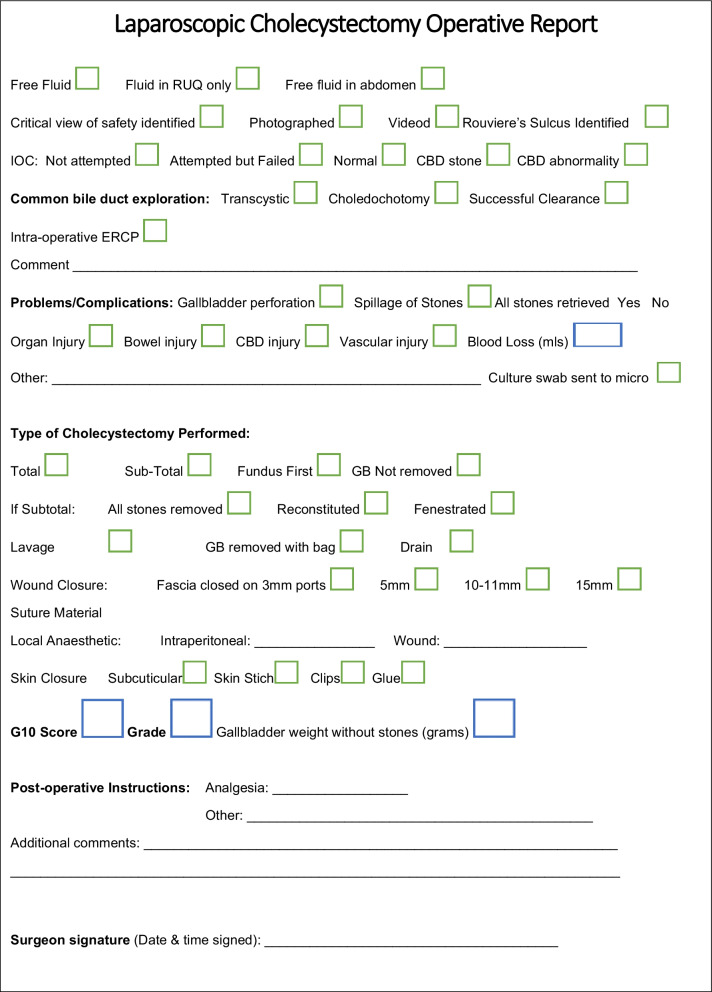
Proposed synoptic operative report for laparoscopic cholecystectomy

Following interrogation of existing safety evidence and previously published templates, a proposed synoptic laparoscopic operative reporting template is shown in Fig. 2. This has been translated into Chinese/Mandarin, French and Arabic (Additional files 1, 2 and 3).

## Discussion

This systematic review identified a paucity of publications dealing with operative reporting of LC. Of the 23 articles with reference to cholecystectomy, only six utilized a data extraction template [9, 13, 20, 23, 31, 42]. Several publications referenced existing generic operative guidelines published by the RCS and RCSI. The DSS guidelines, recommending specific safety steps, have led to many publications on reporting the CVS [44] (Dutch Surgical Society Guidelines 2016). Only six papers dealt with entire operating reporting and both Deal’s and Thomson’s were comprehensive [9, 23].

The mode of recording the operative note was historically paper-based, consisting of a handwritten narrative at the surgeon’s discretion. However, operative note recording is rarely taught to residents. Eichholz et al. in a survey of US Program directors and Borchert, in a UK survey of surgical tutors, both recommended formal training during residency of operative note-writing to improve surgical documentation [6, 46]. Many studies report that both consultants and residents frequently omit some essential surgical elements [42].

St John et al. in a recent prospective study on the documentation in the consent process in general and breast surgery found handwritten forms were associated with a high error rate by omitting key elements compared to a standard template [47]. Paper-based systems have evolved over time to dictated and typed reports which, in many institutions, have now been incorporated into electronic health records. This reflects advances in other areas of medicine such as pathology and radiology where synoptic reporting has improved communication and reduced reporting delays. Two recent meta-analyses by Stogryn et al. and Eryigit et al. compared synoptic versus narrative operative reports across a wide range of surgical disciplines [11, 48]. Both including publications from Harvey and Thomson dealing with LC [23, 31]. Both demonstrated that synoptic reporting was significantly more complete than the narrative one with shorter completion times. Synoptic operative reports, whether hand written, dictated and typed or generated de novo using a computerized template, should ideally be procedure specific. The completeness of operative reports in LC has been shown to be improved by synoptic reporting [9, 20, 23, 31, 42].

An alternative to synoptic reporting in LC was proposed by Stewart et al. [49]. The authors hypothesized that if more attention were paid to the objectives of operative reports, their content would more predictably contain the most relevant information, which might channel thinking in beneficial directions during surgery. Using the method of cognitive task analysis, the authors identified a number of key steps in the performance of LC. By framing the surgeon’s thinking, cognitive task analysis would be expected to reduce operative complications. Stewart and colleagues argued that supplanting a narrative operative report with a synoptic template (with limited free text input) would result in the loss of important information including contextual background. Accordingly, we have added a narrative section at the end of the proposed report to capture other important data that were not captured by the form. In future the checklist should have the option of self-generating a written narrative report.

A key challenge in LC is the identification of the CVS. Although considered an essential surgical safety step many studies have found a lack of proper documentation of the CVS in the operative notes. This concept was reinforced in the study by Wauben et al. who identified that the written operative notes do not adequately represent the actual LC performed often omitting important procedural steps [18]. More than 15 years have elapsed since the Dutch Society of Surgery recommendation on image registration [50]. Even when documented in the notes, the view of the CVS is not always confirmed on video recordings. Most but not all studies found video recording to be more helpful and accurate than photo documentation of the CVS. Eryigit et al. suggested audio addition to enhance accuracy and to improve understanding of decision-making [13]. More recently, Sobba et al. instituted an innovative operative image messaging service in an attempt to establish better agreement among surgeons about obtaining the CVS [10].

There have been no prospective studies or RCTs relating to the type of OR report with patient outcomes. Confining our search to English language only is a limitation of this systematic review and ability to undertake a bias analysis was difficult.

The findings at LC for both elective and emergency surgery can be so variable that many authors have attempted to grade or score the findings. This offers some standardization when trying to assess the outcome of operative strategy and decision making. However, none of the currently reported LC specific templates have incorporated a scoring system. In a previous study, Sugrue et al. introduced the G10 score (and grade of difficulty) to define the status of the gallbladder at surgery for documentation in the operative report [38, 39]. This may facilitate a better understanding of intra-operative events and may lead to improved post-operative care and better patient outcomes but is somewhat cumbersome to calculate.

Ideally the LC operative report we have outlined should be computerized to facilitate database storage and retrieval, and should incorporate drop down menu options with automated calculation of both operative score and grade. Under template headings, such as indications for surgery, a digital drop down would allow the surgeon to choose options including; biliary colic, simple/complex cholecystitis, empyema biliary peritonitis. Linkage of the operative report to the electronic healthcare record has already been demonstrated [34]. Combining audio, video and photographic documentation with the narrative aspect of the synoptic report will enhance the accuracy of record-keeping despite possible elevated costs associated with the creation of digital archives [13]. Jung et al. have promoted the concept of the documentation of safety the creation of a black box, and while not included in Fig. 2 it is an option in further versions of the template [51]. The inclusion of a document safety check list at the start of the procedure will fit with increased need for global safety in surgery [52]. Omission is a potential challenge with any operative report and ten Broek et al. have identified that surgeons may not record adverse intra-op event [53]. In a study of 755 operative reports, they found 6/43 inadvertent enterotomies and 17 of 48 organ injuries were not reported, contributed in part by delays in completing the operative report.

Video recordings of surgical procedures are not new but may be a source of anxiety among surgeons. Efforts by Doyen in the late eighteen-hundreds to introduce cinematography to the teaching of surgery were undoubtedly popular at medical conferences but were harshly criticized by his contemporaries in France, who felt that the integrity of their profession had been compromised [2]. There are also concerns over the potential for medico-legal liability of stored records although such records also offer the potential for a robust defence in these cases. General data protection regulations must be adhered to with all recordings and need to be incorporated in patient consent [54]. The emergence of Surgical Data Science as a specialty in its own right will help us to manage and provide support for ever-expanding hospital data archives, to which synoptic reports and record archives are no exception.

## Conclusion

Our systematic review has identified variable approaches to recording LC operation notes, with limited scientific publications in the area. The proposed new template will have the facility to integrate digitally with hospitals’ medical systems. The translation into many languages by WSES board will encourage global uptake. Synoptic templated operation notes, with inclusion of narrative text and supplemented by audio-visual data will undoubtedly provide the best options for advancing operative care in gallbladder disease in the twenty-first century.

## Supplementary Information

**Additional file 1.** Proposed synoptic operative report for Laparoscopic Cholecystectomy [Chinese/Mandarin translation].

**Additional file 2.** Proposed synoptic operative report for Laparoscopic Cholecystectomy [French translation].

**Additional file 3.** Proposed synoptic operative report for Laparoscopic Cholecystectomy [Arabic translation].

## Data Availability

All data generated or analyzed during this study are included in this published article.

## Abbreviations

BDI: Biliary duct injury
CBD: Common bile duct
CVS: Critical view of safety
DORs: Dictated operative reports
DSS: Dutch society of surgery
IOC: Intra-operative cholangiography
LC: Laparoscopic cholecystectomy
OR: Operating room
MINORS: Methodological index for non-randomized studies
NHS: National health service
PRISMA: Preferred reporting items for systematic reviews and meta-analyses
PROSPERO: Prospective register of systematic reviews
RCT: Randomized controlled trial
RCS Engl: Royal College of Surgeons of England
RCSI: Royal College of Surgeons of Ireland
SORs: Synoptic operative reports
WSES: World Society of Emergency Surgery
