# Supplementary material for: It’s time for a minimum synoptic operation template in patients undergoing laparoscopic cholecystectomy: a systematic review
Source: World J Emerg Surg. 2022 Mar 17;17:15. doi: 10.1186/s13017-022-00411-5 (PMC8928637; doi:10.1186/s13017-022-00411-5)
Supplement: Supplementary file 1 — Additional file 1. Proposed synoptic operative report for Laparoscopic Cholecystectomy [Chinese/Mandarin translation]. [file 13017_2022_411_MOESM1_ESM.pdf]

腹腔镜胆囊切除术手术报告

病人姓名: ____________ 入院日期: __________ 医院编号: _________

日期: ____________ 时间: ____________

年龄: ________ 外科医生: _____________________

性别: _________ 外科助理: ____________________

BMI: _____ 麻醉师: ___________________

ASA 分数: _______ 手术助理护士: __________________

手术详情:

*****如果是, 请输入" Y", 不是请输入 "N" 在 ☐ **,** 请输入数值在 ☐

预防性抗生素☐ 药物: _________ 给药时间: __________

手术指征: _____________________ 择期 ☐ 或 紧急 ☐

手术名称: _____________________

方法: 腹腔镜手术 ☐ 腹腔镜转换开放手术 ☐

如果转换, 转换原因: ____

腹腔入口: Veress ☐ Semi-Open-"Hassan" ☐

直视端口☐ 端口数: ☐

尺寸: ≤4mm ☐ 5mm ☐ 10-11mm ☐ 15mm ☐

手术结果**:** ____

胆囊外的状态: 正常 ☐

其他观察或症状: ____

腹内粘连: 没☐ 右季肋部☐ 右髂窝☐ 扩散粘连☐

胆囊调查结果**:**

大小: 干瘪☐ 正常☐ 膨胀☐

胆囊粘连: 没☐ <50% ☐ >50% ☐ 隐藏☐

是否无需降压也能抓住胆囊 ☐ 需降压胆囊☐

粘液腺囊肿☐ 积脓☐ 瘘管☐

腹腔镜胆囊切除术手术报告

自由流体☐ 流体限制在右季肋部 ☐ 腹部流体 ☐

确认 Critical view of safety ☐ 照片 ☐ 视频 ☐ 确认 Rouviere ‘s Sulcus ☐

胆管造影: 没☐ 尝试但失败☐ 正常☐ 胆总管结石☐ 胆总管异常☐

胆总管探查:

跨囊☐ 胆总管切开术☐ 成功去除胆总管结石 ☐

术中 内鏡逆行胰膽管造影术☐

评诂____

问题/并发症: 胆囊穿破☐ 胆囊结石溢出☐ 所有结石被取出 ☐

器官损伤☐ 肠损伤☐ 胆总管损伤☐ 血管损伤☐ 失血量(mls) ☐

其他: ________________________________ 标本采集☐

胆囊切除术类型:

全胆囊 ☐ 次全 ☐ 眼底优先技术 ☐ **胆囊没取出** ☐

如果次全: 结石完全取出 ☐ Reconstituted ☐ Fenestrated ☐

灌洗☐ 胆囊切除 ☐

伤口缝合闭方式:

筋膜缝合在 3mm 套管针端口☐ 5mm ☐ 10-11mm ☐ 15mm ☐

缝针材料: ____________

局部麻醉: 腹腔 ☐ 手术部位: ☐

缝合伤口: 皮下☐ 皮肤缝合☐ 缝合夹☐ 肤胶☐

G10 分数 ☐ ☐ 级别 ☐ 胆囊重量（处于结石）（克数）☐

术后指示: 静脉输液 24 小时: __________ 止痛方式: ___________

其他: ____________________________

其他评论: ______________________________________

____

外科医生签证 (日期和时间): __________________________
