# Supplementary material for: It’s time for a minimum synoptic operation template in patients undergoing laparoscopic cholecystectomy: a systematic review
Source: World J Emerg Surg. 2022 Mar 17;17:15. doi: 10.1186/s13017-022-00411-5 (PMC8928637; doi:10.1186/s13017-022-00411-5)
Supplement: Supplementary file 2 — Additional file 2. Proposed synoptic operative report for Laparoscopic Cholecystectomy [French translation]. [file 13017_2022_411_MOESM2_ESM.pdf]

Compte Rendu Opératoire Cholécystectomie Laparoscopique

Nom du patient: _____________ Date d'admission: ___________ Hôpital: __________

Date: _____________ Heure: _____________

Age: _________ Chirurgien: _______________________

Sexe: __________ Assistants: ______________________

BMI: _____ Anesthésiste: _____________________

ASA Score: _______ IBODE: ____________________

Détails de la procédure: ***Insérez "O" = Oui, ou "N" = Non dans** ☐ **et des valeurs numériques dans** ☐

Antibioprophylaxie ☐ Type: __________ Moment de l'administration: ___________

Indication chirurgicale: _______________________ Programmé ☐ **ou** Urgence ☐

Opération réalisée: __________________________

Abord: Laparoscopie ☐ Conversion ☐

Si conversion précisez les raisons pour convertir: ____

Insertion de trocarts: Aiguille de Verres ☐ Méthode semi-ouvert-"Hassan" ☐

Trocart inséré sous contrôle visuel ☐ Trocarts: Nombre ☐

Taille: ≤4mm ☐ 5mm ☐ 10-11mm ☐ 15mm ☐

Constatations Opératoires: ____

____

Exploration de la cavité abdominale en dehors de la vésicule biliaire: Normale ☐

Commentaires/Anomalies Organes Abdominaux: ____

Adhérences Intra-Abdominales: Aucune ☐ HD ☐ FID ☐ Diffuses ☐

Vésicule biliaire:

Taille: Recroquevillée ☐ Normale ☐ Distendue ☐

Adhérences à la paroi vésiculaire: Aucune ☐ <50% ☐ >50% ☐ Accès complètement fermé ☐

Possibilité d'attraper la VB sans vidange ☐ VB vidangée ☐

Mucocèle ☐ Empyème ☐ Fistule ☐

Compte Rendu Opératoire Cholécystectomie Laparoscopique

Epanchement ☐ Epanchement seulement en HD ☐ Epanchement péritonéal généralisé ☐

Vue critique de sécurité identifiée ☐ Photographiée ☐ Filmée ☐ Sulcus de Rouvière Identifié ☐

Cholangiographie peropératoire: Non ☐ Echoué ☐ Normale ☐ Calculs VBP ☐ Anomalies VBP ☐

Exploration de la voie biliaire principale: Transcystique ☐ Cholédochotomie ☐ Clairance Complète ☐

CPRE peropératoire ☐

Commentaires ____

Problèmes/Complications: Perforation VB ☐ Calculs intrapéritonéaux ☐ Calculs récupérés en totalité Oui Non

Blessure organes adjacents ☐ Plaie Grêle ☐ Plaie VBP ☐ Plaie vasculaire ☐ Pertes sanguines (ml) ☐

Autre: ____ Prélèvements bactériologiques ☐

Type de Cholécystectomie Réalisée:

Totale ☐ Sub-Totale ☐ Antérograde ☐ VB Laissée en Place ☐

Si Sub-totale: Tous les calculs récupérés ☐ Fermeture Infundibulaire ☐ Fenestration ☐

Lavage ☐ VB extériorisée dans un sac ☐

Fermeture pariétale: Fascia fermé sur trocarts de: 3mm ☐ 5mm ☐ 10-11mm ☐ 15mm ☐

Fils de Suture utilisés

Anesthésique local: Intrapéritonéal: _______________ Pariétal: __________________

Fermeture Cutanée Plan Sous-Cutané ☐ Peau: Points ☐ Agraffes ☐ Colle ☐

Score G10 ☐ ☐ **Grade** ☐ Poids VB sans calculs (grammes) ☐

Consignes Postopératoires: Perfusion intraveineuse 24heures: __________ Analgésie: ________________

Autre: ____

Autres Commentaires: ____

____

Signature du Chirurgien (Date & heure): _____________________________________
