# Supplementary material for: It’s time for a minimum synoptic operation template in patients undergoing laparoscopic cholecystectomy: a systematic review
Source: World J Emerg Surg. 2022 Mar 17;17:15. doi: 10.1186/s13017-022-00411-5 (PMC8928637; doi:10.1186/s13017-022-00411-5)
Supplement: Supplementary file 3 — Additional file 3. Proposed synoptic operative report for Laparoscopic Cholecystectomy [Arabic translation]. [file 13017_2022_411_MOESM3_ESM.pdf]

Arabic Translation by Hani O. Eid and Fikri Abu-Zidan

تقرير جراحي لاستئصال المرارة بالمنظار

اسم المريض: ________________ تاريخ الدخول: ______________ رقم الملف الطبي: _____________

التاريخ: __________ الوقت: _____:_____

العمر: __________ الجراح: __________________

الجنس: __________ المساعد: __________________

مؤشر كتلة الجسم: _____ طبيب التخدير: ________________

نقاط مقياس الجمعية الأمريكية للتخدير: _______ الممرض: __________________

تفاصيل الاجراء الجراحي: أدخل "ن" = نعم، "ل" = لا في ☐ و قيمة عددية في ☐

مضادات حيوية وقائية ☐ النوع: ________________________ وقت الإعطاء: ____________

استطباب الجراحة: ____________________ اختياري ☐ أم طارئ ☐

العملية المجراة: ____________________________________

أسلوب العملية: تنظير ☐ تحويل لفتح جراحي ☐

إذا تم التحويل، سبب التحويل: ______________________________________

الدخول الصفاقي: فيريس ☐ شبه مفتوح "هاسون" ☐

تم إدخال المنفذ تحت الرؤية المباشرة ☐ منافذ الدخول: العدد ☐

المقاس: ≤ 4 مم ☐ 5 مم ☐ 10-11 مم ☐ 15 مم ☐

الموجودات الجراحية العامة:

____

____

تنظير البطن العام خارج المرارة: طبيعي ☐

ملاحظات/ الموجودات الأخرى في البطن: ____

الالتصاقات داخل البطن: لا يوجد ☐ الربع العلوي الأيمن ☐ الحفرة الحرقفية اليمنى ☐ منتشرة ☐

الموجودات المرارية:

الحجم: منكمشة ☐ طبيعية ☐ متمددة ☐

الالتصاقات على المرارة: لا يوجد ☐ < 50% ☐ > 50% ☐ مدفونة ☐

يمكن امساك المرارة دون تخفيف الضغط ☐ تم تخفيف ضغط المرارة ☐

قيلة مخاطية ☐ تقيح المرارة ☐ ناسور ☐

تقرير جراحي لاستئصال المرارة بالمنظار

سائل حر ☐ سائل في الربع العلوي الأيمن ☐ سائل حر في البطن ☐

تم تحديد منظر الأمان بدقة ☐ تم أخذ صورة ضوئية ☐ تم تصويرها بالفيديو ☐ تم تحديد تلم روفيير ☐

تصوير الأقنية الصفراوية أثناء العملية: لم يحاول ☐ لم تنجح المحاولة ☐ طبيعي ☐ حصاة القناة الصفراوية المشتركة ☐

شذوذ في القناة الصفراوية المشتركة ☐

استكشاف القناة الصفراوية المشتركة: عبر القناة المرارية ☐ ببضع قناة الصفراء ☐ تخليص ناجح ☐

تصوير البنكرياس والقنوات الصفراوية بالمنظار أثناء العملية ☐

ملاحظة ____________________

المشاكل/المضاعفات: انثقاب المرارة ☐ انسكاب الحصاوي ☐ تم استخراج جميع الحصاوي **نعم لا**

إصابة عضو ☐ إصابة الأمعاء ☐ إصابة القناة الصفراوية المشتركة ☐ إصابة وعاء دموي ☐ فقدان الدم (مل) ☐

أخرى: ____________________ ارسال مسحة الزراعة للمختبر ☐

نوع إجراء استئصال المرارة:

كلي ☐ جزئي ☐ القاع أولا ☐ لم يتم إزالة المرارة ☐

إذا كان جزئيا: تم إزالة جميع الحصى ☐ إعادة تكوين ☐ تثقيب ☐

غسيل ☐ ازيلت المرارة داخل كيس ☐

إغلاق الجرح: تم إغلاق الصفاق على منافذ 3 مم ☐ 5 مم ☐ 11-10 مم ☐ 15 مم ☐

مواد خياطة

مخدر موضعي: داخل جوف البطن : ____________________ الجرح: ____________________

إغلاق الجلد: تحت الجلد ☐ غرزة الجلد ☐ دبابيس ☐ غراء ☐

نقاط G10 ☐ ☐ **الدرجة** ☐ وزن المرارة بدون حصوات (جرام) ☐

تعليمات ما بعد الجراحة: محاليل وريدية ل 24 ساعة: ____________________ مسكنات: ____________________

أخرى: ____________________

ملاحظات أخرى: ____________________

____

توقيع الجراح (تاريخ ووقت التوقيع): ____________________
